# Proactive approaches to preventing postpartum depression in non-depressive pregnant women: a comprehensive scoping review

**DOI:** 10.3389/fgwh.2025.1497740

**Published:** 2025-04-09

**Authors:** Nga Thi Nguyen, Supa Pengpid

**Affiliations:** 1 Department of Health Education and Behavioral Sciences, Faculty of Public Health, Mahidol University, Bangkok, Thailand; 2 Department of Epidemiology- Biostatistics and Demography, Faculty of Public Health, Hue University of Medicine and Pharmacy, Hue University, Hue, Vietnam

**Keywords:** postpartum depression, intervention, prevention, pregnant women, scoping review

## Abstract

**Introduction:**

Postpartum depression is a significant global health challenge that affects mothers, infants, and families. Although various preventive strategies show promise, comprehensive reviews evaluating interventions among pregnant women without a clinical diagnosis of depression remain limited. This scoping review aims to identify and synthesize the existing evidence on proactive postpartum depression prevention programs initiated during pregnancy.

**Methods:**

Following PRISMA guidelines for scoping reviews, we systematically searched PubMed and Scopus, supplemented by manual reference reviews. Our search strategy combined terms related to postpartum depression, pregnancy, and preventive interventions. Studies were included if they evaluated interventions conducted during pregnancy, targeting women without a clinical diagnosis of depression, and assessed PPD outcomes using established diagnostic criteria or validated screening tools. Only English-language articles published between 2013 and 2023 were considered.

**Results:**

A total of 49 studies met the inclusion criteria. Interventions were categorized into nine themes: psychoeducation (*n* = 18), home visits (*n* = 6), cognitive behavioral therapy (CBT) (*n* = 6), mindfulness (*n* = 6), exercise (*n* = 4), dietary supplements (*n* = 3), interpersonal therapy (IPT) (*n* = 4), consultation (*n* = 1), and inhalation aromatherapy (*n* = 1). Psychoeducational and mindfulness-based interventions consistently reduce PPD risk, particularly when delivered in structured, theory-driven formats and incorporating family support. Digital CBT interventions demonstrated limited effectiveness due to lower engagement, while home-visit and consultation-based interventions were effective when integrated into existing maternal healthcare despite scalability challenges. Exercise and dietary supplement interventions yielded inconsistent outcomes, indicating that factors such as adherence, duration, and intensity are crucial determinants of effectiveness.

**Conclusion:**

Various proactive interventions are available to prevent PPD, and this scoping review systematically maps the different strategies used and their outcomes. Proactive, theory-based, and multi-component interventions, particularly psychoeducational and mindfulness programs, demonstrate promising potential. Future research should emphasize evaluating long-term outcomes, optimizing digital engagement strategies, and developing culturally tailored models to enhance scalability and accessibility across diverse populations, including low-resource settings.

## 1 Introduction

Mental health is a critical public health issue, with the World Health Organization (WHO) reporting that one in eight people worldwide experience mental health disorders (1). Notably, 71% of individuals with psychosis do not receive treatment, and depression accounts for 28.9% of mental disorders, making it the second leading cause of Global Years of Healthy Life Lost due to disability (2). Postpartum depression (PPD) is a major maternal and child health concern, characterized by symptoms such as sadness, hopelessness, loss of interest, sleep disturbances, fatigue, and poor concentration (3, 4). A systematic review found that approximately 17.22% of women experience PPD, which can significantly affect their quality of life, disrupt family dynamics, and impair child development if left untreated (5). PPD can also interfere with maternal-infant bonding and the mother's ability to care for her baby, leading to breastfeeding difficulties and developmental challenges (6–8). Despite the prevalence of PPD, over 75% of affected women, particularly in low- and middle-income countries, do not receive treatment, with only 13.6% actively seeking help (9). Barriers to care include financial constraints, lack of awareness about perinatal depression, a shortage of trained healthcare professionals, and the social stigma associated with mental health disorders (10, 11). Access to psychiatric care remains limited, especially in developing countries. Given these challenges, PPD prevention is a critical priority to improve maternal and infant well-being.

Although the exact causes of PPD remain unclear, multiple risk factors contribute to its onset, including hormonal changes, psychosocial and social stressors (6). Specific risk factors include a history of depression, prenatal depression, intimate partner violence, concerns about the child's health, breastfeeding difficulties, low family and spousal support, lack of awareness, and cultural confinement practices (12–15). Conversely, self-efficacy and social support have been identified as protective factors against PPD (16, 17). Bandura's Self-Efficacy Theory suggests that individuals with strong self-efficacy are better equipped to manage stress and depressive symptoms (18). Additionally, James S. House identified four types of functional support—emotional, instrumental, informational, and appraisal—that can help mitigate PPD risk. Addressing these risk and protective factors during pregnancy through targeted interventions may be key to PPD prevention (19, 20). Previous reviews have explored various preventive measures, including nutrition, physical activity, mindfulness, and psychological and psychosocial interventions. While some studies have compared these approaches, they often included both pregnant and postpartum women, some of whom were already experiencing depression (21–26). A review of psychosocial and psychological interventions suggested that early pregnancy interventions can effectively reduce PPD risk in at-risk women (21). More recently, an umbrella review by Motrico et al. examined multiple PPD prevention strategies (27). However, there remains a lack of research focusing exclusively on pregnant women without clinical depression, making it difficult to assess the true effectiveness of preventive interventions in this population. Including depressive participants in such studies may confound results, as their use of medications or psychological therapy could either overestimate or underestimate intervention effects.

Despite these research efforts, a comprehensive mapping of PPD prevention programs exclusively targeting non-depressive pregnant women is still lacking. To address this gap, we conducted a scoping review to identify and synthesize available evidence on intervention programs aimed at preventing PPD in non-depressive pregnant women. This review examines the scope and characteristics of these interventions, highlights key initiatives, and explores their implications for clinical practice and policy development to enhance maternal mental health care.

## 2 Methods

This scoping review follows the Preferred Reporting Items for Systematic Reviews and Meta-Analyses (PRISMA) guidelines for scoping reviews to systematically map interventions aimed at preventing PPD during pregnancy.

### 2.1 Study selection

Studies were selected via electronic databases, including Pubmed and Scopus, which included multiple additional databases (e.g., Embase, PsycINFO, Cochrane) that would increase redundancy due to significant overlap in indexed journals. The combined use of PubMed and Scopus facilitated an efficient and systematic literature search, reducing duplication while ensuring the inclusion of diverse intervention studies.

The search strategy included the following terms: The search strategy included the following terms: (“postpartum period”[MeSH Terms] OR “postpartum” OR “postnatal” OR “puerperal” OR “peripartum period”[MeSH Terms] OR “peripartum” OR “prenatal” OR “antenatal” OR “pregnancy”[MeSH Terms] OR “intrapartum” OR “pregnant women”[MeSH Terms]) AND (“depression, postpartum”[MeSH Terms] OR “depression postpartum” OR “depressive disorder”[MeSH Terms] OR “depression”[MeSH Terms] OR “depress*”) AND ((prevent* OR “intervention*”)).

Additionally, systematic reviews and meta-analyses on PPD prevention were manually searched, and reference lists from relevant randomized controlled trials (RCTs) were reviewed. Supplementary Table 1 provides search strings and detailed results from each database. Given the increasing role of digital health in prevention, we focused on studies published between 2013 and 2023. Only English-language articles were included.

### 2.2 Eligibility criteria

This scoping review included original studies evaluating interventions for preventing PPD in pregnant women without a clinical diagnosis of depression or severe depressive symptoms. To ensure a comprehensive assessment, both pharmacological and non-pharmacological interventions were considered without restrictions on intervention type. Studies were eligible if PPD was assessed at least once postpartum using established diagnostic criteria, including the International Classification of Diseases, 11th Revision (ICD-11), the Diagnostic and Statistical Manual of Mental Disorders, Fifth Edition (DSM-5), or validated screening tools such as the Edinburgh Postnatal Depression Scale (EPDS), Beck Depression Inventory (BDI) (3, 4, 28, 29). EPDS is widely used to screen for depressive and anxiety symptoms during or after pregnancy, while BDI measures depression severity, aiding healthcare providers in screening and monitoring treatment progress (28, 29). Studies that included both depressed and non-depressed perinatal women at baseline were considered only if separate results were reported for non-depressed participants. The selection process adhered to the PICO framework: P: Pregnant women without a clinical diagnosis of depression or severe depressive symptoms; I: Preventive intervention programs; C: Care as usual or other comparison conditions (including active controls, enhanced usual care, or no comparison group; O: Postpartum depression as the primary outcome. Studies were included if they met the following criteria: (1) the intervention was conducted during pregnancy, (2) participants were pregnant women without depression at baseline, (3) the study was published in English, and (4) the publication period was between 2013 and 2023. Exclusion criteria included cohort, case-control, and cross-sectional studies, case reports, case series, reviews, letters, protocols, book chapters, and opinion papers.

### 2.3 Data charting process

After retrieving studies from PubMed and Scopus, records were imported into EndNote 20, where duplicates were automatically and manually removed. The EndNote library was shared with the research team for independent screening of titles, abstracts, and full texts. Two researchers extracted data independently using a shared Excel spreadsheet, categorizing articles by intervention type through discussion and consensus.

**Figure 1:**
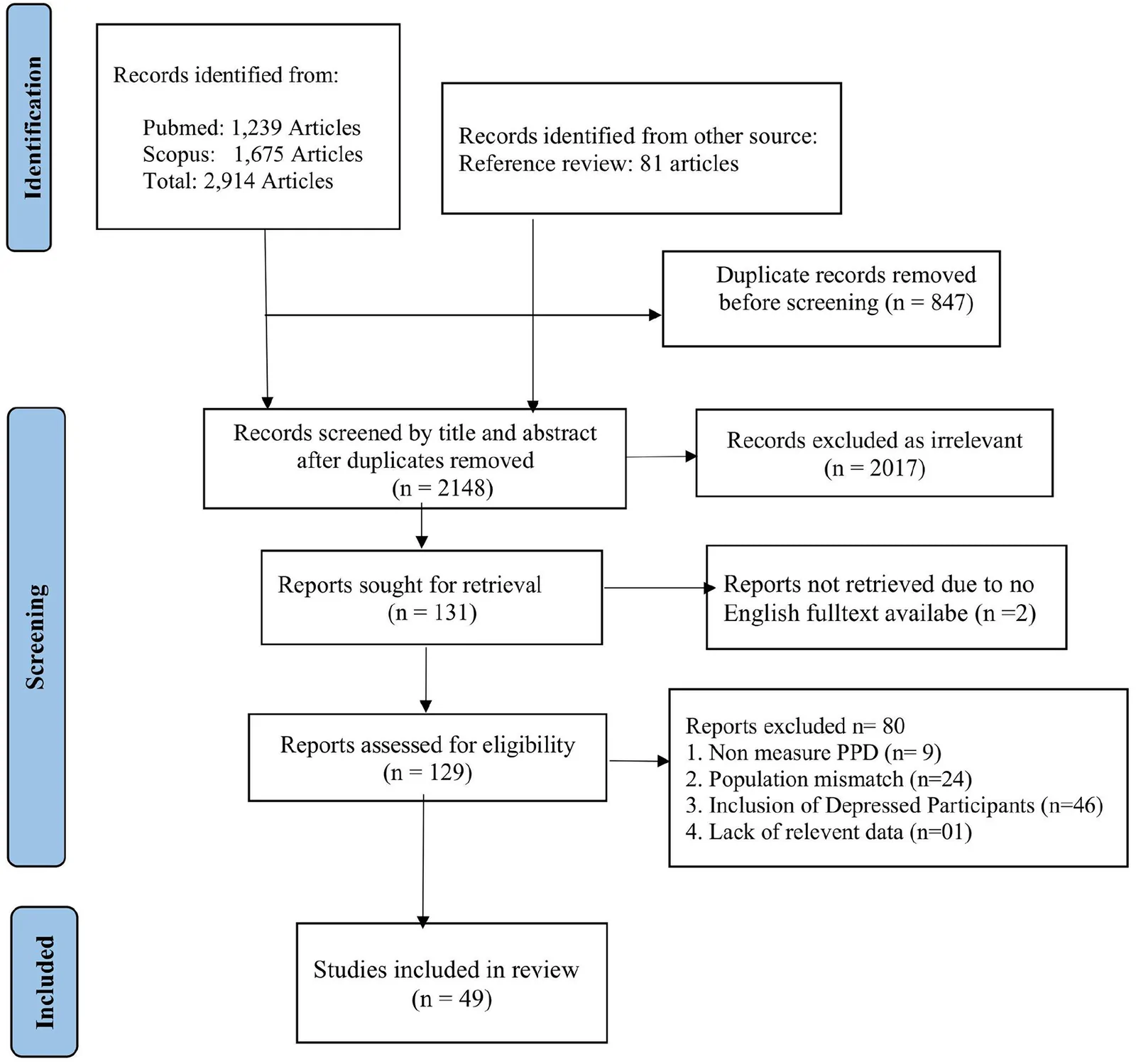
PRISMA flow diagram of the selection process of studies on preventing postpartum depression from pregnancy.

Figure 1 presents the PRISMA flow diagram detailing our study selection process. We identified a total of 2,914 records from PubMed (1,239 articles) and Scopus (1,675 articles), along with 81 additional records from reference reviews. After removing 847 duplicate records, 2,148 records were screened by title and abstract. Of these, 2,017 records were excluded as irrelevant, leaving 131 full-text articles to be retrieved. Two full-text articles were not obtained due to the unavailability of an English version, resulting in 129 full-text articles assessed for eligibility. Among these, 80 articles were excluded for the following reasons: nine articles focused solely on prenatal depression; 24 involved populations other than pregnant women; 46 included participants with depression at baseline; and one lacked data specific to pregnant women. Ultimately, 49 studies met our inclusion criteria and were included in this review.

### 2.4 Collating, summarising, and reporting results

The papers were organized by theme and summarised without considering the evidence's strength. This study gives insights into global early prevention programs for PPD. This review maps global PPD prevention programs, identifying intervention categories without assessing study quality. The aim is to explore intervention approaches, target populations, and contextual variations to assess their potential effectiveness across different settings.

## 3 Results

### 3.1 Characteristics of the included study

This scoping review included 49 studies (see Table 1) evaluating interventions to prevent PPD in pregnant women without a clinical diagnosis of depression. These studies were conducted in high-income (*n* = 26) (31, 32, 34, 36–40, 45, 46, 48, 50, 53, 57, 60, 63–65, 68–70, 75–77, 79) and upper-middle-income (*n* = 22) (30, 35, 41–44, 47, 51, 54–56, 58, 59, 61, 62, 66, 67, 71–74, 78) countries, with one study spanning 23 countries (33). Most studies employed RCT design, while others used quasi-experimental and pre-post-experimental approaches. Sample sizes ranged from 30 to 5,017 participants (31).

**Table 1:** Summary of prevention programs on postpartum depression from pregnancy.

No.	Author (Year)	Study setting	Study design	Population/sample size	Research instruments	Postpartum assessment	Type of intervention	Comparison group	Key finding
1	Boran et al. (2023) (30)	Turkey	RCT	Healthy pregnant women (*n* = 88)	EPDS, STAI, PRAQ-R, PHQ-9	4-6 weeks	CBT^a^ Zoom platform, 5 sessions, 60 min each	Treatment-as-usual	Depression rates at baseline and final assessments were not different as measured by EPDS and PHQ-9
2	Nishi et al. (2022) (31)	Japan	RCT	Healthy pregnant women (*n* = 5017)	New MDE onset, K6 scale	3 months	CBT^a^ Luna Baby App, Finished by 32 weeks, flexible access	General online information	No overall preventive effect; effective for subthreshold distress.
3	Le et al. (2020) (32)	Spain	Non-experimental design	Pregnant women at high risk of PPD (*n* = 30)	Patient health questionnaire	3 and 6 months	CBT direct class, 3 sessions, 2 h each	None	Reduced depressive symptoms, feasible and acceptable
4	Barrera et al. (2015) (33)	23 countries	RCT	Healthy pregnant women (*n* = 111)	CED-S EDPS major depressive episode screener	3 months, 6 months	CBT^a^ Online intervention, Flexible access	An electronic version of a PPD information brochure	The intervention was more beneficial than the control for women with higher prenatal depression scores, but not for those at low risk
5	Bittner et al. (2014) (34)	Germany	RCT	Pregnant women at risk of PPD (*n* = 118)	EPDS ≥10	3 months	CBT Direct class, 8 sessions, 90 min each	Routine prenatal care	Effectiveness for groups with EPDS ≥10 at baseline, no effectiveness for groups with EPDS<10
6	Ramezani et al. (2017) (35)	Iran	RCT	Healthy pregnant women (*n* = 85)	EPDS	15 days	Cognitive-behaviour counseling direct counseling, 7 sessions, 1.5 h each	Routine prenatal care	Reduced maternity blues & depression significantly
7	Tandon et al. (2021) (36)	USA	Cluster randomised design	Healthy pregnant women (*n* = 824)	Quick inventory of depressive symptomatology self-report	12-24 weeks	Home visit 6 sessions, 60–90 min, delivered by mental health professionals & paraprofessional staff	Usual home visiting	Reduced depressive symptoms from baseline to 24 weeks postpartum, but there were no significant differences between usual care and either intervention group
8	Tandon et al. (2022) (37)	USA	Quasi-experimental study	Pregnant women at high risk of PPD (*n* = 1,229)	Beck depression scale	6 months	Home visit 12 sessions, 15–20 min, delivered by lay home visitors	Usual home visiting	Reductions in perceived stress and depressive symptoms
9	Kenyon et al. (2016) (38)	UK	RCT	Pregnant women with social risk factors (*n* = 1,324)	EPDS maternal-infant bonding	6 weeks	Home visit flexible, until postpartum, deliverd by pregnancy outreach workers	Standard maternity care	No significant differences were seen in the mean EPDS for all the women recruited but reduced depression in high-risk subgroup of PPD
10	Cooper et al. (2016) (39)	UK	RCT	Pregnant women at high-risk of PPD (*n* = 165)	EPDS	8, 8 weeks, 12,18 months	Home visit 2 prenatal, 9 postpartum visits, delivered by Research health visitors & NHS health visitors	Standard maternity care	No significant effect on postpartum depression or maternal behaviors
11	Tandon el al. (2018) (40)	USA	RCT	Pregnant women with non-major PPD (*n* = 120)	The Beck's depression inventory-II	3 months and 6 months	Home visit 12 sessions, delivered by home visitors	Routine care	No significant difference in depressive symptoms was observed at 3 months postpartum; a significant difference emerged at 6 months
12	Öztoprak et al. (2023) (41)	Turkey	RCT	Primiparous mother at low risk of PPD (*n* = 64)	EPDS, PSAS, self-care power scale, MPQoL	10th day, 6 weeks, 3 months	Home visit 3 home visits, phone follow-ups	Standard postpartum care	Improved self-care, quality of life, and reduced depression/anxiety
13	Y.Sun et al. (2021) (42)	China	RCT	Pregnant women at risk of PPD (*n* = 168)	EPDS	6 weeks	Mindfulness^a^ Mobile app training, 8 weeks of training	8-week regular WeChat health consultations.	Reduced depressive symptoms and anxiety; improved affect, especially in nulliparous women
14	Wang et al. (2023) (43)	China	RCT	Healthy pregnant women (*n* = 104)	EDPS	3 days and 42 days	Mindfulness Direct class, and practice at home, 4 weeks	Received an online childbirth education course with a recorded video on the WeChat applet for 21 days	Reduced depressive symptoms, anxiety, Fear of childbirth; increased life satisfaction
15	Leng et al. (2023) (44)	China	RTC	Pregnant women at high risk of stress (*n* = 75)	EPDS obstetric outcomes, mindfulness	4–6 weeks	Mindfulness^a^ Application-based self-learning, 8 weekly sessions	Web-based education	Reduced depression scores, lower C-section risk, higher Apgar scores
16	Hassdenteufel et al. (2023) (45)	Germany	RCT	Pregnant women at high risk of PPD (EPDS >9) (*n* = 460)	EPDS, STAI, PRAQ-R, PHQ-9	1 month and 5 months	Mindfulness^a^ Online intervention, 8 weekly sessions	Care as usual	No significant reduction in postpartum depression between groups over time. Reduced pregnancy- and birth-related anxiety, and improved mindfulness
17	Pan et al. (2019) (46)	Taiwan	RCT	Pregnant women and their husbands (*n* = 74)	EPDS	3 months	Mindfulness Direct intervention, 8 weeks	Usual prenatal care	Significant reduction in depression/stress postpartum
18	Guo et al. (2020) (47)	China	RCT	Pregnant women at high risk of PPD (*n* = 284)	EPDS	3 months and 1 year	Mindful self-compassion^a^ Online intervention, 10 h total	Care as usual	Reduced depression, improved infant outcomes
19	Arakawa et al. (2023) (48)	Japan	RCT	Pregnant women (*n* = 734)	EPDS, self-efficacy, loneliness	3 months	Health consultation^a^ Unlimited online consultations	Usual care	Lower postpartum depressive symptoms, increased self-efficacy, reduced loneliness
20	Cordero et al. (2018) (49)	Spain	RCT	Healthy pregnant women (*n* = 129)	EPDS	Between 4 and 6 weeks	Exercise Aquatic exercise, moderate physical activity, 3 times/week, 17 weeks	Routine prenatal care	Significant differences were found between the exercise and control groups in EPDS scores and in BMI among overweight/obese participants
21	Navas el al. (2021) (50)	Spain	RCT	Healthy pregnant women (*n* = 320)	EPDS	1 month	Exercise Aerobic exercise with controlled heart rate, 3 times/week, 5 months	Usual prenatal care	Reduced postpartum anxiety/depression
22	Coll et al. (2019) (51)	Brazil	RCT	Healthy pregnant women (*n* = 649)	EPDS	3 months	Exercise Direct fitness instruction, 3 times/week, 16 weeks	Routine care	No significant reduction; possible effect of non-compliance
23	Mohammadi et al. (2015) (52)	Iran	RCT	Healthy pregnant women (*n* = 127)	EPDS	1 month, 2 months	Exercise Home-based stretching and breathing exercises, 3 times/week, 20–30 min	Inactive control	Improved psychological well-being; no effect on weight gain
24	Shimpuku et al. (2022) (53)	Japan	Quasi-experimental design	Healthy pregnant women (*n* = 221)	EPDS	1 month, 3 months	Educational program Direct class with videos, 2 h, one-time	Standard care + leaflet	Reduced EPDS scores, increased parenting confidence
25	Zhao et al. (2021) (54)	China	RCT	Pregnant women at high risk of PPD (EPDS >9) (*n* = 182)	EPDS breastfeeding outcomes	3 days, 3 months	Educational program Direct class with midwives, 4 sessions, 60 min each	Usual prenatal care	Reduced postpartum depression, improved breastfeeding
26	Mohammadi et al. (2021) (55)	Iran	RCT	Healthy pregnant women (*n* = 64)	Beck's depression	1 week and 1 month	Educational program Direct class, 8 sessions, 2 h each	Routine prenatal care	Reduced postpartum depression, increased vaginal delivery
27	Beydokhti et al. (2021) (56)	Iran	RCT	Pregnant women at low risk (*n* = 137)	EPDS	4–6 weeks	Educational program Direct class, 4 sessions, 60–90 min	Routine prenatal care	Reduced postpartum depression scores
28	Scorza et al. (2020) (57)	USA	Quasi- experimental design	Healthy pregnant women and poverty (*n* = 60)	EPDS	6 weeks and 16 weeks	Educational program and mindfulness Direct class, 3 sessions, 150 min total	Enhanced usual care	Reduced symptoms at 6 weeks; no reduction in clinical disorders
29	Moshki et al. (2014) (58)	Iran	Pre-post experimental design	Healthy pregnant women (*n* = 230)	EPDS	4 weeks	Educational program, 3 workshops, 4 h each	Routine care	Reduced postpartum depression scores
30	Zhao et al. (2019) (59)	China	RCT	Pregnant women with complications of pregnancy and at low- risk of PPD and their husbands (*n* = 352)	EPDS	3 days, 42 days	Psycho-educational Program Direct class, 6 sessions, 2 h each	Usual care	Reduced postpartum depression scores
31	Haga et al. (2019) (60)	Norway	RCT	Healthy pregnant women (*n* = 1,324)	EPDS	Ga 37 weeks, 6 weeks, 3 months, 6 months postpartum	Educational program* Web-based self-learning, 44 sessions over 11.5 months	Standard prenatal care	Reduced depressive symptoms postpartum at 6 week significantly
32	Sanaati et al. (2018) (61)	Iran	RCT	Pregnant women at high risk of PPD and their husbands (*n* = 189)	EPDS	6 weeks	Lifestyle educational Program Direct class, 4 sessions, 60–90 min	Routine care	Reduced depression/anxiety scores significantly
33	Zhao et al. (2018) (62)	China	RCT	Pregnant women with complications of pregnancy, high risk of PPD and their husbands (*n* = 352)	EPDS	3 days, 42 days	Psycho-educational program Direct class, 6 sessions, 90 min each	Usual care	Reduced depression, better birth outcomes
34	Collado et al. (2014) (63)	Spain/France	RCT	Pregnant women at risk of PPD (*n* = 184)	EPDS	4 weeks	Educational program Direct class, during second trimester	Standard antenatal care	Improved birth outcomes; no significant overall reduction in PPD
35	Cauli et al. (2019) (64)	Italy	RCT	Pregnant women (*n* = 318)	EPDS	2 months	Educational program Direct class, different intervention for high-risk, low-risk and no-risk groups	Routine prenatal care	Intervention program is effective among high-risk group, not in no risk group
36	Shorey et al. (2019) (65)	Singapore	RCT	Pregnant women and their husbands (*n* = 118 couples)	EPDS	2 days, 1 month, 3 months	Educational program Direct class, 4-week postpartum support	Routine perinatal care	Reduced PPD, anxiety; improved bonding and parenting self-efficacy
37	Çankaya et al. (2020) (66)	Turkey	RCT	Healthy pregnant women (*n* = 120)	DASS	6–8 weeks	Educational program Direct class, 2 weeks	Routine prenatal care	Reduced depression/anxiety; increased self-efficacy; higher vaginal birth rates
38	Ahmadpour et al. (2022) (67)	Iran	RCT	Healthy pregnant women (*n* = 106)	EPDS	4–6 weeks	Educational program	Routine hospital care	Reduced postpartum depression and PTSD, improved childbirth experience
39	Shorey et al. (2023) (68)	Singapore	RCT	Pregnant women and their husbands (*n* = 200 couples)	EPDS	1 month, 2,4, 6, 9, 12 months	Psychoeducational program^a^Mobile app & peer support, 2 sessions	Usual care	Increased perceived social support; no significant reduction of depression score
40	Nakajima et al. (2023) (69)	Japan	RCT	Pregnant women and their husbands (*n* = 37)	EPDS	1 month, 3 months	Education program	Routine prenatal care	Reduced postpartum depression; enhanced marital relationship postpartum
41	Ahrne et al. (2023) (70)	Sweden	A quasi-experimental design	Pregnant women (*n* = 145)	EPDS	2-months	Education program	Standard antenatal care	Improved emotional well-being, particularly reduced depressive symptoms
42	Khodadad et al. (2021) (71)	Iran	RCT	Pregnant women at risk (*n* = 86)	EPDS	1.5 months	Supplements (Vitamin 6)	Control	Significantly reduced postpartum depression
43	Vaz et al. (2017) (72)	Brazil	RCT	Healthy pregnant women (*n* = 60)	EPDS	4–6 weeks	Supplements (Omega3)	Control	No overall effect; benefits for women with prior depression
44	Sousa et al. (2023) (73)	Brazil	RCT	Healthy pregnant women (*n* = 60)	EPDS	2 weeks, 1-month, 4-months, 6- months	Supplement (omega-3)	Placebo (Olive oil)	No significant overall differences; earlier symptom reduction in Omega-3 group
45	Kianpour et al. (2018) (74)	Iran	RCT	Healthy pregnant women (*n* = 90)	EPDS	2 weeks, 6 weeks	Inhalation Aromatherapy (lavender and rose)	7 drops of odorless sesame seed oil	Significantly reduced depression at 2 & 6 weeks postpartum
46	Zlotnick et al. (2016) (75)	USA	RCT	Pregnant women at high risk of PPD (*n* = 205)	DSM-V	3, 6, 12 months	Interpersonal therapy	Usual care	Lower depression rate postpartum compared to controls within 6 months significantly
47	Phipps et al. (2013) (76)	USA	RCT	Healthy adolescent pregnancy (*n* = 106)	KID-SCID	6 weeks, 3 months, 6 months	Interpersonal therapy	Used the baby basics book	New cases of PPD reduced without significance
48	Phipps et al. (2020) (77)	USA	RCT	Healthy adolescent pregnancy (*n* = 250)	KID-SCID	3 months, 6 months, 12 months	Interpersonal therapy	Used the baby basics book	No significant differences of new cases reduction between two groups; possibly influenced by community support
49	Ngai et al. (2022) (78)	China	RCT	Pregnant women and their husbands (*n* = 455)	EPDS	6 weeks, 6 months	Interpersonal therapy	Usual care	Reduced depressive symptoms at two measurement times

CED-S, center for epidemiologic studies depression scale; CBT, cognitive behavioral therapy; RCT, randomized clinical trial; EPDS, Edinburgh postnatal depression scale; WHO-CIDI 3.0, WHO composite international diagnostic interview; MINI, the mini-international neuropsychiatric interview; DSM-V, the diagnostic and statistical manual of mental disorders; PPD, postpartum depression; KID-SCID, the structured clinical interview for DSM-IV childhood diagnoses; SCID, structured clinical interview for DSM disorders.

a Online.

Interventions were categorized into nine themes: psychoeducation (*n* = 18), home visits (*n* = 6), cognitive behavioral therapy (CBT) (*n* = 6), mindfulness (*n* = 6), exercise (*n* = 4), dietary supplements (*n* = 3), interpersonal therapy (IPT) (*n* = 4), consultation (*n* = 1), and inhalation aromatherapy (*n* = 1) (see Table 2). Most studies targeted healthy pregnant women, while others focused on high-risk groups, such as those with social risk factors, adolescent pregnancyor pregnancy complications. Eight studies included both pregnant women and their partners, emphasizing family involvement in PPD prevention. The EPDS was the most commonly used outcome measure, alongside the Beck Depression Inventory and the Patient Health Questionnaire. The contents of each intervention are presented in Supplementary Table 2.

**Table 2:** Summary of intervention types, modes of delivery, and reported outcomes in preventing postpartum depression.

Intervention type (number of articles)	Delivery mode	Number reporting positive outcomes	Best for	Challenges
Psychoeducation (*n* = 18)	In-person, digital, hybrid	16	General & high-risk women	Requires cultural adaptation
CBT (*n* = 6)	In-person, digital	2	High-risk populations	Digital versions had low adherence
Mindfulness (*n* = 6)	In-person, apps	5	Cultures with postpartum recovery traditions	Engagement varies by format
Home Visits (*n* = 6)	Community-based	3	High-risk women in structured healthcare systems	Scalability in low-resource settings
Exercise (*n* = 4)	Supervised & self-guided	2	Women with moderate physical activity levels	Adherence challenges
IPT (*n* = 4)	Group & partner-based	2	High-risk pregnant women	Requires structured delivery
Dietary Supplements (*n* = 3)	Vitamin B6, omega-3	1	Women with dietary deficiencies	Requires better adherence tracking

CBT, cognitive behavioral therapy; IPT, interpersonal therapy.

### 3.2 Characteristics of the intervention prevention

#### 3.2.1 Cognitive behavioral therapy intervention

Six studies integrated CBT interventions into prenatal care to help pregnant women manage anxiety, depression, and stress through psychoeducation, behavioral activation, and problem-solving. These interventions targeted both healthy pregnant women and those at high risk of PPD, delivered via in-person group sessions or digital platforms (Zoom, apps, and websites). Digital programs included a five-session Zoom-based CBT intervention (30), self-paced CBT modules via the Luna Baby App (31) and an interactive CBT-based website for mood regulation (33). In-person approaches for high-risk women included a three-session CBT program focusing on thought restructuring, an eight-session group-based CBT intervention addressing anxiety and postpartum adjustment (34) and seven cognitive-behavioral counseling sessions incorporating solution-focused techniques (35).

The effectiveness of CBT-based interventions in preventing PPD varies based on the delivery mode**.** Online CBT interventions targeting healthy pregnant women demonstrated limited effectiveness, with RCTs assessing digital CBT adaptations, including the Thinking Healthy Program (30) and a smartphone-based CBT program (31) showing no significant reductions in depressive symptoms compared to control groups. Similarly, an eight-session internet-based CBT intervention by Barrera et al. failed to show superiority over standard informational materials at 3 and 6 months postpartum (33).

Conversely, in-person CBT interventions were more effective, particularly in high-risk populations. The Mothers and Babies Program (32) and other structured onsite CBT interventions demonstrated significant reductions in depressive symptoms, especially among participants with elevated baseline EPDS scores (34). A study comparing CBT and solution-focused counseling reported significantly lower EPDS scores in both intervention groups at 15 days postpartum, with no significant difference between therapeutic approaches (35).

Overall, in-person CBT interventions were effective, particularly among high-risk pregnant women, whereas digital CBT programs had limited impact on healthy pregnant women. The lack of direct engagement in digital CBT may contribute to lower adherence and reduced effectiveness. These findings highlight the need for hybrid models that integrate interactive features, real-time therapist support, and personalized guidance to improve digital CBT engagement and efficacy in PPD prevention.

#### 3.2.2 Mindfulness-based interventions

Mindfulness interventions aimed to enhance self-awareness, emotional regulation, and stress management in pregnant women through meditation, breathing exercises, and mindful movement**.** These interventions were delivered via mobile apps, online platforms, and in-person hospital training, increasing accessibility and adaptability for diverse populations. Digital approaches included mobile app-based mindfulness programs with guided exercises (42, 44, 45) and WeChat-supported training with childbirth education (43). In-person formats featured mindful self-compassion training (47) and group-based mindfulness sessions involving both pregnant women and their husbands (46), reinforcing social and emotional support during pregnancy.

Effectiveness varied based on delivery method and target population**.** Among six studies evaluating mindfulness interventions**,** five reported significant reductions in depressive symptoms, particularly in high-risk pregnant women. Mobile-delivered mindfulness interventions with psychotherapist support (54) and smartphone-based mindfulness programs (78) effectively reduced depressive symptoms, demonstrating the importance of guided facilitation in digital approaches. Similarly, an RCT with healthy pregnant women demonstrated significant improvements compared to usual care, suggesting that mindfulness can be beneficial even for low-risk populations (46). Interventions combining mindfulness with self-compassion techniques showed sustained benefits up to one year postpartum**,** while a four-week mindfulness-based childbirth and parenting program significantly reduced depression, anxiety, and fear of childbirth (43). However, one study found no significant differences in depressive symptoms with an app-based mindfulness program compared to usual care, highlighting the need for structured engagement strategies in digital mindfulness interventions (63).

Mindfulness interventions were largely effective in preventing PPD**,** with in-person and therapist-supported digital formats yielding the most significant benefits. Interventions that combined self-compassion techniques or social support showed greater long-term effectiveness. However, standalone app-based mindfulness programs without therapist interaction had limited impact**,** suggesting that active engagement and guided facilitation may be crucial for optimizing digital mindfulness interventions. These findings emphasize the need for hybrid models integrating digital and in-person mindfulness approaches, ensuring accessibility and sustained maternal mental health benefits.

#### 3.2.3 Interpersonal therapy (IPT)

IPT interventions aimed to support pregnant women at risk of PPD by strengthening social support, communication skills, and relationship management. These programs utilized group discussions and individual therapy to provide tailored support, addressing emotional and interpersonal challenges. Delivery formats varied, including small-group IPT sessions (75), couple-based IPT programs (78) and adolescent-focused IPT interventions (76, 77) ensuring accessibility across different populations

Four studies evaluated the impact of IPT on PPD prevention, with two focusing on adult pregnancy and two targeting pregnant adolescents. IPT intervention for adult pregnant women with postnatal booster sessions effectively reduced PPD incidence in the US and China at six months postpartum (75, 78). Zlotnick et al. (75) examined IPT's impact on high-risk pregnant women, engaging 205 women, while Ngai et al. (78) studied 455 healthy first-time parents, both demonstrating significant reductions in postpartum depressive symptoms. Conversely, IPT interventions for adolescent pregnancies showed no significant preventive effect. Phipps et al. (76, 77) assessed the same IPT program across two studies (2013, 2020), with the first including 106 primiparous adolescents and the second 250 general pregnant adolescents. Neither study demonstrated IPT's effectiveness in preventing PPD among adolescent populations, suggesting that traditional IPT models may not fully address the unique psychosocial stressors of teenage pregnancies.

IPT demonstrated effectiveness in reducing PPD risk when applied to adult populations, particularly high-risk women and first-time parents. Couple-based IPT interventions were particularly beneficial, reinforcing partner support and emotional resilience during pregnancy. However, IPT programs for adolescent pregnancies failed to prevent PPD, highlighting the need for age-specific adaptations that account for developmental, social, and structural challenges unique to adolescent mothers. These findings suggest that tailored intervention strategies—incorporating peer support, parental involvement, and adolescent-focused mental health frameworks—may enhance the efficacy of IPT in younger populations.

#### 3.2.4 Home visit programs

Home-visit interventions provided personalized psychoeducation, emotional support, and lifestyle guidance to pregnant women, with variations in structure, duration, and professional involvement. These programs were integrated into maternity care systems in the UK (38, 39), the US (36, 37, 40), and Turkey (72), demonstrating different approaches to maternal mental health support. In the UK, home visits were embedded into routine maternity care. Pregnancy Outreach Workers offered flexible support (39) and NHS health visitors conducted structured sessions focusing on maternal mental health and bonding (39). In the US, home-visit interventions had a stronger emphasis on therapy-based support, incorporating individualized cognitive-behavioral therapy (CBT) sessions (40), group-based emotional regulation programs (36) and frequent stress-management visits (37)In Turkey, home visits were supplemented with digital check-ins and postpartum follow-up calls to enhance accessibility (72).

Three studies by Tandon et al. in the US evaluated the Mothers and Babies 1-1 (MB) program, which integrates cognitive-behavioral intervention into home visits. The first RCT demonstrated that women receiving MB-based home visits had significant reductions in depressive symptoms at three and six months postpartum compared to the control group (40). The second study compared the effectiveness of mental health professionals and paraprofessionals delivering the intervention. While there was no significant difference between intervention and usual care groups, paraprofessional-led visits were as effective as those led by mental health professionals, suggesting the feasibility of task-shifting in home-visit programs (36). The third quasi-experimental study found that the intervention, when delivered by lay-home workers, significantly reduced Beck Depression Inventory (BDI) scores, with higher attendance linked to greater reductions in depressive symptoms (37).

Additional home-visit interventions were evaluated in the UK and Turkey. In the UK, Cooper et al. (39) examined a program focused on enhancing mother-infant relationships but found no significant effect on PPD prevention. Another RCT by Kenyon et al. (38) assessed a home-visit intervention for nulliparous women under 28 weeks gestation with social risk factors; while the intervention group showed a reduction in mean EPDS scores, there was no statistically significant difference from the control group. In Turkey, Öztoprak et al. (72) evaluated a nurse-led home-visit counseling program, finding that three home visits significantly reduced PPD symptoms among participants.

Home-visit interventions demonstrated mixed effectiveness, with structured CBT-based programs yielding the most promising results. The US-based MB program showed significant reductions in depressive symptoms, particularly when delivered by paraprofessionals or lay-home workers, supporting task-shifting as a viable strategy. In contrast, UK-based home visits without integrated mental health components showed limited impact on PPD prevention. The nurse-led program in Turkey successfully reduced PPD symptoms, indicating that trained healthcare professionals providing home-based psychological support may enhance intervention efficacy. These findings suggest that home-visit programs are most effective when they incorporate structured psychological interventions, particularly CBT-based approaches or counseling by trained professionals. The scalability of these programs, especially in low-resource settings, may benefit from task-sharing models and digital follow-ups to increase accessibility and sustainability.

#### 3.2.5 Psychoeducation/education interventions

Psychoeducational interventions aim to enhance maternal knowledge, self-efficacy, emotional well-being, and resilience by incorporating theoretical frameworks such as Bandura's Social Cognitive Theory, Self-Efficacy Theory, Health Locus of Control Theory, Social Support Theory, Bowlby's Attachment Theory, and the PRECEDE-PROCEED Model (55, 56, 58, 65, 68). These interventions are delivered through in-person classes, digital platforms, mobile health (mHealth) apps, and hybrid models, often involving partners to enhance social support.

Among the 18 studies assessing psychoeducational interventions for PPD prevention, 12 targeted pregnant women only, while six included both pregnant women and their husbands. Effectiveness varied across different intervention designs. Programs focusing on healthy pregnant women primarily emphasized childbirth preparation, stress management, and postpartum adaptation. Notable programs, including the Self-Efficacy-Based Psychoeducation Program (55) and the Health Locus of Control Psychoeducation Program (61), demonstrated significant reductions in depressive symptoms when compared to control groups. Similarly, individualized prenatal education focused on breastfeeding, birth planning, and psychological preparation (67) resulted in improved maternal confidence and lower EPDS scores postpartum. Digital education interventions, such as the Mamma Mia Web-Based Program (60) and the Online Maternal Mental Health Consultation Program (48), allowed for self-paced learning and interactive engagement, though some digital-only interventions without interactive support showed inconsistent results (65).

For pregnant women at higher risk of PPD, interventions were designed to build emotional resilience, improve coping mechanisms, and facilitate early detection of mood disturbances. The CBT Psychoeducation Program (54) the PRECEDE-PROCEED Psychoeducation Model (56) and the Lifestyle-Based Psychoeducation Program (61) integrated structured cognitive-behavioral and psychoeducational techniques, resulting in greater self-regulation and lower postpartum depressive symptoms. Similarly, psychoeducation programs tailored for pregnant women with medical complications- such as the High-Risk Pregnancy Mental Health Program (59, 62) and the Psychosomatic Antenatal Education Program (63) effectively reduced anxiety and improved birth preparedness.

Partner-inclusive interventions demonstrated higher effectiveness in reducing PPD rates, emphasizing the role of family support in maternal mental health. Programs such as the Postpartum Support Program (65) and the Couple-Based Pregnancy Program (69) reinforced partner involvement, resulting in lower EPDS scores and improved postpartum adaptation. Studies found that when partners actively participated, maternal depressive symptoms significantly decreased compared to interventions targeting only pregnant women (42, 59). Additionally, lifestyle-based psychoeducation (67) showed that couple-focused education had a greater impact than individual-based programs. However, digital partner-based interventions, such as the Supportive Parenting App (65) had mixed results, likely due to limited engagement or inadequate personalization.

Hybrid models integrating in-person sessions with digital follow-ups demonstrated high effectiveness in sustaining maternal engagement and knowledge retention. Programs such as the Home Visit with Digital Follow-Up Program (41) and the Three-Step Perinatal Psychoeducation Model (68) provided continuous maternal support beyond traditional clinical settings, reinforcing educational retention and adherence. The Multidisciplinary Psychosocial Intervention (64) introduced a stratified approach, offering psychotherapy, counseling, or general psychoeducation based on PPD risk levels, showing positive results in reducing depressive symptoms in high-risk women.

Psychoeducational interventions demonstrated strong effectiveness in reducing postpartum depressive symptoms, particularly when grounded in self-efficacy, social support, and emotional regulation theories. Programs that incorporated partners or family members had greater long-term benefits, highlighting the importance of shared postpartum adaptation. Digital interventions increased accessibility and flexibility, but engagement and interaction levels influenced their effectiveness. Hybrid models combining face-to-face education with digital reinforcement were among the most effective strategies, ensuring long-term maternal support and continuous learning. These findings suggest that structured, theory-based psychoeducational interventions, especially those incorporating family support, interactive digital tools, and long-term engagement strategies, are essential for effective PPD prevention. Future research should focus on optimizing digital components, ensuring accessibility in diverse populations, and evaluating long-term mental health outcomes to strengthen the sustainability of these interventions.

#### 3.2.6 Exercise-based interventions

Exercise-based interventions have been implemented to promote moderate physical activity during pregnancy, aiming to improve physical and mental well-being. These interventions commonly incorporate aerobic activities, strength training, and flexibility exercises, delivered either in structured group settings or as guided home-based programs. Aquatic-based interventions, such as a 17-week aerobic and strength-training program (49) and a five-month structured aquatic aerobic exercise program with controlled heart rate monitoring (50), improved cardiovascular fitness, and significantly reduced depressive symptoms compared to controls. However, not all interventions were effective. A 16-week moderate-intensity exercise program by Coll et al. showed no significant reduction in PPD incidence. At the same time, home-based stretching and breathing intervention by Mohammadi et al. was ineffective, likely due to poor adherence (51, 52).

Exercise-based interventions offer a promising, non-pharmacological approach to PPD prevention, but effectiveness is highly dependent on supervision and adherence. Structured, supervised programs, mainly aquatic and group-based land interventions, demonstrated the most consistent benefits, emphasizing the role of social engagement and guided facilitation in the success of the intervention. In contrast, home-based and self-guided exercise programs had lower effectiveness, highlighting the need for strategies to improve adherence, such as digital tracking tools or personalized coaching.

#### 3.2.7 Dietary supplements

Three studies examined the role of nutritional supplementation in preventing PPD, focusing on vitamin B6 and omega-3 fatty acids. Vitamin B6 supplementation (40 mg twice daily from the 28th week of pregnancy until one month postpartum) significantly reduced depressive symptoms at 1.5 months postpartum compared to controls, suggesting a potential role in maternal mood regulation (71). In contrast, omega-3 supplementation (16 weeks of fish oil capsules during the third trimester) showed no significant effect on depressive symptoms in healthy pregnant women, indicating limited impact on PPD prevention (72, 73).

These findings suggest that vitamin B6 may help reduce postpartum depressive symptoms, while omega-3 supplementation appears ineffective. Further research is needed to determine the optimal dosage, timing, and target populations for supplementation.

#### 3.2.8 Inhalation aromatherapy and consultation

Aromatherapy interventions utilize natural relaxation techniques to reduce stress and anxiety. Kianpour et al. (44) provided lavender and rose water essential oils for pregnant women to inhale before sleep from 38 weeks of gestation to 6 weeks postpartum, with phone call follow-ups ensuring adherence. Compared to an odorless placebo, aromatherapy significantly reduced EPDS scores, suggesting its potential role in lowering PPD risk.

mHealth consultation interventions provide on-demand medical support to address health concerns and promote maternal well-being. Arakawa et al. evaluated a healthcare service via the LINE messaging app, offering 10-minute consultations through voice calls, text, or video, with unlimited access from pregnancy to postpartum. The intervention significantly reduced the risk of elevated postpartum depressive symptoms, highlighting mHealth's potential in PPD prevention (48).

Across interventions, psychoeducational and mindfulness-based programs consistently reduced PPD risk, especially when delivered in structured, theory-driven formats and incorporating family support. In contrast, digital CBT interventions for healthy pregnant women exhibited limited effectiveness, likely due to reduced engagement, while in-person CBT interventions showed significant benefits among high-risk groups. Home-visit programs yielded mixed results: those integrating psychological components (e.g., the Mothers and Babies program) were effective, whereas programs lacking such components showed limited impact. Exercise-based interventions and dietary supplements produced inconsistent outcomes, underscoring that factors such as adherence, intervention intensity, and duration are critical. Additional modalities—such as inhalation aromatherapy and mHealth consultations—demonstrated promising results in reducing depressive symptoms (see Table 2).

## 4 Discussion

This scoping review identified and synthesized available evidence on intervention programs aimed at preventing postpartum depression (PPD) among pregnant women without a clinical diagnosis of PPD. A total of 49 studies were included, representing diverse geographic, methodological, and thematic variations in interventions. The interventions fell into nine key categories: educational/psychoeducational interventions, CBT, mindfulness, exercise, home-visit programs, dietary supplements, IPT, inhalation aromatherapy, and consultation-based approaches. The findings demonstrate that while several interventions show promise in reducing PPD risk, variations in delivery formats, target populations, and methodological rigor contribute to mixed evidence regarding their overall effectiveness.

Educational and psychoeducational interventions were the most commonly studied preventive approaches, with 18 studies examining their efficacy. These interventions typically focused on improving maternal knowledge, self-efficacy, and emotional preparedness for childbirth and postpartum. They also trained pregnant women on how to cope with the difficulties in childcare or to seek support from their family members. 16/18 interventions demonstrated significant reductions in depressive symptoms, particularly when interventions were tailored to high-risk populations or involved partners (54–56, 58). A systematic review by Dennis et al. (21) concluded from four trials that education programs had no preventive effect on PPD, which is not in line with our review. Differences in the content and duration of programs between reviews can explain this inconsistency. Moreover, with four trials included in the review of Dennis, there may not be enough evidence to conclude the effectiveness of education and psychoeducation intervention among pregnant women. Therefore, it is crucial to conduct a detailed and up-to-date review of educational programs to determine the overall impact.

WHO recommends CBT as an evidence-based psychosocial intervention to support perinatal women with depressive symptoms at the community level (80). Six studies assessed the efficacy of CBT in preventing postpartum depression (PPD). The findings indicate that none of the three digital CBT interventions demonstrated significant efficacy, whereas two of the three face-to-face interventions significantly reduced the incidence of PPD in pregnant individuals identified as being at high risk, despite the absence of a clinical diagnosis of major depressive disorder at baseline. These results align with a meta-analysis by Muira et al. (20), which found no significant difference in PPD incidence between application-based CBT and control groups, with substantial heterogeneity in the reduction of Edinburgh Postnatal Depression Scale (EPDS) scores. Moreover, a comprehensive review of seven systematic reviews on CBT interventions concluded that initiating CBT during pregnancy may reduce the risk of developing PPD, although effect sizes varied (81). These findings highlight the clinical relevance of early screening for perinatal individuals at high risk of PPD as a preventive strategy. While internet-based interventions offer a scalable and cost-effective approach, modifications may be necessary to optimize patient engagement, adherence, and therapeutic efficacy.

Mindfulness-based interventions are cognitive-behavioral therapeutic approaches that cultivate present-moment awareness through structured meditation practices, including breathwork, body scanning, auditory perception, and cognitive diffusion techniques. These interventions facilitate emotional regulation and enhance self-compassion, enabling pregnant individuals to develop adaptive coping mechanisms for stressors associated with pregnancy, labor, and the postpartum period (44–47). Among six studies evaluating the efficacy of mindfulness interventions, five reported a significant reduction in depressive symptoms. Furthermore, a systematic review by. Trapani et al. (22) demonstrated that mindfulness-based programs may effectively prevent PPD within the first three months postpartum.

The impact of home-visit programs on PPD prevention remains variable. Of the six studies included, two reported a statistically significant reduction in depressive symptoms. The *Mothers and Babies* 1-on-1 program in the United States demonstrated effectiveness when administered by trained home visitors or lay health workers to pregnant individuals identified as being at high risk for PPD (37, 40). However, small-group interventions targeting the general obstetric population did not yield comparable results (36). In contrast, a home-visit program implemented in the United Kingdom failed to produce significant reductions in perinatal depressive symptoms. Notably, baseline EPDS scores were unavailable in both UK-based studies, limiting the ability to quantify intervention efficacy (38, 39). Given the heterogeneity in study design, participant characteristics, and intervention modalities, the overall effectiveness of home-visit programs remains inconclusive. Moreover, home-visit interventions were implemented in the countries with home-visit programs available that support perinatal women. Therefore, it is challenging for countries without this program.

Interpersonal therapy (IPT) is an evidence-based psychosocial intervention that has demonstrated efficacy in the prevention of postpartum depression (PPD), particularly among individuals with identifiable risk factors. IPT aims to enhance interpersonal functioning and strengthen social support networks by addressing role transitions, resolving interpersonal conflicts, and processing grief—factors that are particularly salient during the perinatal period. Consistent with the therapeutic principles of IPT, a study conducted in China that incorporated spousal involvement, as well as another study targeting high-risk pregnant individuals, reported significant effectiveness (75, 78). However, our review found that IPT-based interventions were ineffective among healthy adolescent pregnant individuals in two studies conducted in the United States. Unlike older pregnant individuals, adolescents often experience reduced autonomy in their interpersonal relationships and social environments. Many remain financially and emotionally dependent on their families, which may limit the effectiveness of interventions centered on interpersonal role transitions (82). Moreover, adolescent pregnancies are frequently unintended, and young mothers may encounter heightened levels of social stigma, isolation, and parental oversight, further influencing the impact of their social support systems (83). These unique psychosocial challenges may attenuate the effectiveness of IPT-based interventions in this population, necessitating the development of tailored approaches that address the distinct needs of adolescent mothers.

The role of physical activity in PPD prevention remains controversial. Of four studies evaluating exercise interventions, two reported a significant reduction in EPDS scores between intervention and control groups. These findings align with a meta-analysis indicating that aerobic exercise is beneficial for both the prevention and treatment of PPD, despite considerable heterogeneity in outcomes (84). Physiologically, moderate-intensity aerobic exercise stimulates endorphin release and regulates neuroendocrine pathways, contributing to improved mood, sleep quality, self-awareness, and self-esteem while alleviating symptoms of anxiety and depression (85, 86). Consequently, individualized exercise regimens, including frequency, intensity, and duration, should be considered when designing PPD prevention and management strategies.

Additional prevention modalities reviewed in this study include dietary supplementation, inhalation aromatherapy, and psychological counseling. Evidence suggests that vitamin B6 supplementation may confer a protective effect against PPD, aligning with findings from previous reviews (24, 87). However, the assessment of adherence through plasma biomarker analysis rather than self-reported intake should be considered to improve methodological rigor. Moreover, a digital perinatal consultation program in Japan demonstrated effectiveness in reducing perinatal depressive symptoms; however, its implementation required substantial involvement from healthcare providers, including physicians and nurses, for continuous monitoring and follow-up (48). While such a model may be viable in high-resource settings, its feasibility in low- and middle-income contexts warrants further investigation.

## 5 Implications of the review

Our review highlights several implications for both clinical practice and future research in preventing PPD among pregnant women without a clinical diagnosis of depression. We identified nine key intervention programs with varying impacts, with education and psychoeducation interventions emerging as the most common and consistently effective strategies. Integrating structured, theory-based psychoeducation into routine prenatal care, particularly through targeted depression screenings, could enhance accessibility and cost-effectiveness (88). Programs based on Bandura's Self-Efficacy Theory, Health Locus of Control Theory, and Social Support Theory effectively improve maternal confidence, coping skills, and social support networks (55, 65). Additionally, family involvement, especially partner participation, enhances maternal mental health outcomes, yet remains underutilized, warranting further research into optimizing family-centered approaches (89). Similarly, mindfulness-based interventions, delivered both in-person and online, have demonstrated strong effectiveness in settings such as China and Taiwan, where cultural practices like “doing the month” support postpartum recovery (44, 46, 47). These findings suggest that mindfulness-based programs may be particularly beneficial in cultures that emphasize postpartum confinement and structured rest periods. However, our review also identifies a gap in the effective integration of technology in delivering CBT and mindfulness interventions. While digital health tools offer scalable and cost-effective solutions, existing technology-driven programs, such as app-based CBT, have shown limited effectiveness compared to in-person modalities (30, 31, 35). Integrating interactive features, real-time therapist support, and hybrid in-person and digital models could enhance effectiveness.

Moreover, the predominance of short-term outcome assessments, methodological heterogeneity, and the underrepresentation of low-resource settings limit the generalizability of current findings. Future research should prioritize long-term evaluations, standardize intervention protocols, and explore innovative digital solutions to enhance the accessibility, sustainability, and effectiveness of PPD prevention strategies across diverse populations. Expanding research to include culturally and contextually relevant interventions in low-resource settings is particularly important, given the existing disparities in maternal mental health care access (90).

## 6 Limitations

Our study is not without limitations. As a scoping review, it prioritizes breadth over depth, providing a qualitative overview rather than an in-depth analysis. Nevertheless, it successfully fulfills our objective of offering a comprehensive snapshot of intervention programs aimed at preventing PPD in women. Additionally, our inclusion criteria were limited to English-language papers, which may have led to the exclusion of culturally specific interventions published in other languages. Lastly, the broad scope of our research, encompassing a wide array of interventions, resulted in considerable data heterogeneity, rendering meta-analysis impractical. Consequently, a scoping review was the most appropriate approach for this study.

## 7 Conclusion

There are several proactive interventions that can be implemented to prevent PPD, and this scoping review systematically outlines the various strategies employed and their outcomes. This comprehensive scoping review demonstrates that proactive, multi-component interventions, particularly psychoeducational and mindfulness-based programs, offer significant promise for preventing postpartum depression among non-depressive pregnant women. However, variations in delivery mode, participant adherence, and contextual factors contribute to mixed effectiveness across intervention types. Future research should focus on long-term outcomes, optimize digital engagement strategies, and develop culturally tailored, scalable models that integrate family support into routine maternal healthcare. Such efforts will be critical for advancing global maternal mental health and reducing the burden of postpartum depression.

## Data Availability

The original contributions presented in the study are included in the article/Supplementary Material, further inquiries can be directed to the corresponding author.

## Supplementary material

Supplementary Table 1 Search terms in PubMed and Scopus.

Supplementary Table 2 Contents of prevention programs on postpartum depression from pregnancy.
