# Correction: Proactive approaches to preventing postpartum depression in non-depressive pregnant women: a comprehensive scoping review

**DOI:** 10.3389/fgwh.2026.2003962

**Published:** 2026-10-01

**Authors:** Nga Thi Nguyen, Supa Pengpid

**Affiliations:** 1 Department of Health Education and Behavioral Sciences, Faculty of Public Health, Mahidol University, Bangkok, Thailand; 2 Department of Epidemiology- Biostatistics and Demography, Faculty of Public Health, Hue University of Medicine and Pharmacy, Hue University, Hue, Vietnam

**Keywords:** postpartum depression, intervention, prevention, pregnant women, scoping review

**Acknowledgments**

Adding/removing text

In the acknowledgements, the following statement was not included in the published version and is now added to recognize the scholarship support received from Mahidol University:

“This research project was partially supported by scholarships for Ph.D. student from Mahidol University.”

The original version of this article has been updated.

