# Supplementary material for: Proactive approaches to preventing postpartum depression in non-depressive pregnant women: a comprehensive scoping review
Source: Front Glob Womens Health. 2025 Apr 9;6:1497740. doi: 10.3389/fgwh.2025.1497740 (PMC12014592; doi:10.3389/fgwh.2025.1497740)
Supplement: Supplementary Table 1 — Search terms in PubMed and Scopus. [file Table1.docx]

Supplementary 1

**Table 1:** Search terms in PubMed and Scopus

| **PubMed**: | |
| --- | --- |
| **Search terms** | **Items found** |
| #1. Pregnancy  ("postpartum period"[MeSH Terms]) OR (postpartum[Title/Abstract]) OR (postnatal[Title/Abstract]) OR (puerperal[Title/Abstract]) OR ("peripartum period"[MeSH Terms]) OR (peripartum[Title/Abstract]) OR (prenatal[Title/Abstract]) OR (antenatal[Title/Abstract]) OR ("pregnancy"[MeSH Terms]) OR ("pregnancy"[Title/Abstract]) OR (intrapartum[Title/Abstract]) OR ("pregnant women"[MeSH Terms]) OR ("pregnant women"[Title/Abstract]) | 1,341,195 |
| #2. Depression:  ("depression, postpartum"[MeSH Terms]) OR ("depressive disorder"[MeSH Terms]) OR ("depression" [MeSH Terms]) OR (depress*[Title/Abstract]) | 668,549 |
| #3. Prevention:  ((prevent*[Title/Abstract]) OR ((intervention*[Title/Abstract])) | 3,265,709 |
| #4. Design  ((("RCT"[Title/Abstract] OR "randomized control trial*"[Title/Abstract] OR "trial*"[Title/Abstract] OR "before after study*"[Title/Abstract] OR "quasi-experimental study*"[Title/Abstract]) NOT "comment"[Publication Type]) NOT ("protocol"[Title] OR "protocols"[Title])) | 1,438,613 |
| #5. Combination of #1 AND #2 AND #3 AND #4  (((("postpartum period"[MeSH Terms]) OR (postpartum[Title/Abstract]) OR (postnatal[Title/Abstract]) OR (puerperal[Title/Abstract]) OR ("peripartum period"[MeSH Terms]) OR (peripartum[Title/Abstract]) OR (prenatal[Title/Abstract]) OR (antenatal[Title/Abstract]) OR ("pregnancy"[MeSH Terms]) OR ("pregnancy"[Title/Abstract]) OR (intrapartum[Title/Abstract]) OR ("pregnant women"[MeSH Terms]) OR ("pregnant women"[Title/Abstract])) AND (("depression, postpartum"[MeSH Terms]) OR ("depressive disorder"[MeSH Terms]) OR ("depression" [MeSH Terms]) OR (depress*[Title/Abstract]))) AND (((prevent*[Title/Abstract]) OR (intervention*[Title/Abstract])))) AND (((("RCT"[Title/Abstract] OR "randomized control trial*"[Title/Abstract] OR "trial*"[Title/Abstract] OR "before after study*"[Title/Abstract] OR "quasi-experimental study*"[Title/Abstract]) NOT "comment"[Publication Type]) NOT ("protocol"[Title] OR "protocols"[Title]))) AND ((english[Filter]) AND (2013:2023[pdat])) | **1239** |
| **Scopus** | |
| **Search terms** | **Items found** |
| #1. Perinatal period:  TITLE-ABS-KEY ( "postpartum period" OR "postpartum" OR "postnatal" OR "puerperal" OR "peripartum period" OR peripartum OR prenatal OR antenatal OR pregnancy OR intrapartum OR "pregnant women" ) | 1,581,897 |
| #2. Depression:  TITLE-ABS-KEY ( "postpartum depression" OR "Postnatal depression" OR "depressive disorder" OR depression OR depress ) | 1,011,719 |
| #3. Prevention:  TITLE-ABS-KEY ( prevent OR preventing OR preventive OR intervention ) | 11,905,566 |
| # 4 Design  TITLE-ABS-KEY ( RCT OR "randomized control trial" OR "trial" OR "before after study" OR "quasi-experimental study" AND NOT comment AND NOT "Cross-sectional study" AND NOT case- AND control AND NOT cohort AND NOT ( protocol OR protocols ) ) | 4,575,803 |
| #5. Combination of #1 AND #2 AND #3 AND #4  TITLE-ABS-KEY ( ( "postpartum period" OR "postpartum" OR "postnatal" OR "puerperal" OR "peripartum period" OR peripartum OR prenatal OR antenatal OR pregnancy OR intrapartum OR "pregnant women" ) AND ( "postpartum depression" OR "Postnatal depression" OR "depressive disorder" OR depression OR depress ) AND ( prevent OR prevention OR preventive OR intervention ) AND ( rct OR "randomized control trial" OR "trial" OR "before after study" OR "quasi-experimental study" AND NOT comment AND NOT "Cross-sectional study" AND NOT case- AND control AND NOT cohort AND NOT ( protocol OR protocols ) ) ) AND PUBYEAR > 2012 AND PUBYEAR < 2024 | **1,675** |
