# Supplementary material for: Proactive approaches to preventing postpartum depression in non-depressive pregnant women: a comprehensive scoping review
Source: Front Glob Womens Health. 2025 Apr 9;6:1497740. doi: 10.3389/fgwh.2025.1497740 (PMC12014592; doi:10.3389/fgwh.2025.1497740)
Supplement: Supplementary Table 2 — Contents of prevention programs on postpartum depression from pregnancy. [file Table2.docx]

***Supplementary 2***

**Table 2:** Contents of prevention programs on postpartum depression from pregnancy

| **No.** | **Authors** | **Type of intervention** | **Activities and content delivery**  **in intervention group** | **Activities**  **In Control group** | **Mode of information delivery** | **Program delivery** | **Duration** |
| --- | --- | --- | --- | --- | --- | --- | --- |
| 1 | P. Boran et al.  (30) | CBT | The intervention combined routine antenatal sessions and a one-hour Thinking Healthy Program (THP) session.  The THP intervention, based on CBT, provided participants with psychoeducation, behavioral activation, problem-solving strategies, and group support. | The routine antenatal classes offered instruction on pregnancy, labor, and infant care, as well as fundamental details about PPD and how to identify and seek help. | Zoom- Platform training for groups of pregnant women | Antenatal nurse | 5 sessions x 60 min/session within 4 days  Finished before birth. |
| 2 | Nishi et al. (2022) (31) | CBT | The intervention concluded with general information about mental health during pregnancy, the development of the fetus, and six modules of CBTs: (1) Psychoeducation, (2) Case formulation based on a cognitive-behavioral model, (3) Behavioral activation, (4) Self-compassion, (5) Mindfulness, (6) Problem-solving. | General information about mental health during pregnancy and the development of the fetus via the Luna Baby application. | Online therapy with the Luna Baby application | The research team designed the intervention content (Medical Doctor ad PhD) | - Finished by 32 weeks of pregnancy  - Each session takes 5 minutes to complete.  - No limited time of access |
| 3 | Le et al. (2020) (32) | CBT | Contents of the intervention were delivered into three modules:  (1) Thoughts: increasing helpful thoughts and decreasing harmful thoughts,  (2) Activities: increasing pleasant activities,  (3) People Contacts: increasing helpful and decreasing harmful interpersonal interactions.  (3 plus) The labor and delivery process | No control group. | Direct class | Clinical psychologist and Obstetrician for the last session. | 3 sessions x 2hours/session. |
| 4 | Barrera et al. (2015) (33) | CBT | The intervention's content followed the Mama Bebe program in Spain. It consisted of 8 weekly CBT psychoeducational group sessions that taught women mood-regulation skills to prevent perinatal depression. Then, these contents were modified for the online intervention program. Participants were feedback via worksheets on the website. | An electronic version of a PPD information brochure contains symptom descriptions and prevalence rates on perinatal depression, debunking PPD myths, sources on how to seek support in the event of escalating symptom. | Online intervention by using the website | Fully automated self-help approach | Many times, as women wanted. |
| 5 | Bittner et al. (2014) (34) | CBT | The contents of the group CBT program focused on pregnancy and self-assurance training; problem-solving training; anxiety symptoms in pregnancy and prevention and treatment of pregnancy-related anxiety; depressive symptoms in pregnancy and intervention strategies and challenges in future parenthood; baby blues and puerperal period. | Usual prenatal care | Direct class. | Trained clinical psychologist | 8 sessions x 90 min/session. |
| 6 | Ramezani et al. (2017) (35) | Cognitive-behaviour counselling | The intervention program consisted of two technique interventions: The cognitive-behavioral counseling group and the solution-focused counseling.  - The cognitive-behavioral counseling group included an introduction to maternity blues and postpartum depression and cognitive and behavioral concepts; an explanation of the role of thoughts on emotions, the impact of people’s thoughts on mood, body, and action and the thoughts of depressed people; training on depression-prevention activities such as relaxation and mother and baby skin-to-skin contacts. Additionally, participants were assigned homework to document their thoughts and feelings.  - The solution-focused counseling included the introduction of maternity blues and postpartum depression and a review of the solutions to the problems. The researcher also gave homework to the group of pregnant women. | Usual prenatal care | Direct counseling with pregnant women group. | The midwife had been trained in counseling for two years. | - The cognitive behavioral counseling group: 4 sessions x 1.5 hours/sec  - The solution-focused counselling:3 sessions x 1.5 hours/sec |
| 7 | Tandon et al. (2021) (36) | Home visit | Six sessions introduced the importance of managing one’s mood and CBT content related to pleasant activities, identifying helpful and unhelpful thought patterns and introducing strategies for reframing unhelpful thoughts; promoting positive social interactions | Usual home visit program in the US. | Direct visit at home with groups of 3 pregnant women. | Mental Health professionals provided one group, and one group was provided by Paraprofessional staff. | 6 sessions x 60- 90 min/session. |
| 8 | Tandon et al. (2022) (37) | Home visit | The 12-session mother and baby 1-on-1 program included introducing the stress management framework, engaging in pleasant activities, identifying and modifying unhelpful thought patterns, and addressing aspects of contact with other people. | Usual home visits program in the US. | Home visits delivered the information directly to individual pregnant women. | Lay home visitors | 12 sessions x 15-20 min/session |
| 9 | Kenyon et al. (2016) (38) | Home visit | Home visits were integrated into the community midwifery teams. The objectives were to encourage women to attend antenatal appointments, make healthy lifestyle choices, provide social/emotional support, and help ensure that benefits, housing difficulties, and mental health problems were managed. In the postnatal period (to 6 weeks postpartum), POWs also provided breastfeeding and advice about infant care. | Standard UK maternity care | Home visits | Lay support or Pregnancy Outreach Workers | - The unknown number of visits.  - Started from pregnancy to postpartum. |
| 10 | Cooper et al. (2016) (39) | Home visit | Three principal elements: First, supportive counseling was provided to encourage the women to express their feelings in a non-judgemental and supportive context. Second, specific strategies were employed to sensitize the mothers to their infants’ characteristics. Finally, specific help was provided to the mothers in managing infant behavioral problems [i.e., sleeping, feeding, crying) | Usual care. | Home visits. | One group delivered by research health visitors; one group delivered intervention by trained National Health Service health visitors | 2 times before and 9 after delivery, lasted until 16 weeks of postpartum. |
| 11 | Tandon el al. (2018) (40) | Home visit | Mother and baby 1-on-1 delivered by home visitors consisted of 12 sessions. The content of the program included introducing the stress management framework, engaging in pleasant activities, identifying and modifying unhelpful thought patterns, and describing aspects of contact with other people. | Usual home visit program in the US | Direct intervention for individual pregnant women. | Home visitors | 12 sessions x 15-20 min/session |
| 12 | Öztoprak et al. (2023) (41) | Home visit | Training and counseling about:  1. Birth preparation training: signs of labor, preparation of maternity bag, and delivery  2. Postpartum care: bleeding management, excretion, sleep and rest, and breast care. Baby care: umbilical cord care, breastfeeding, and flatulence management.  3. Family planning, sexuality, and pediatric vaccines. | The control group received an educational booklet. | Home visits were appointments by phone calls and messages. | Nurse | Three home visits with five short messages and four phone calls from 36 weeks of gestation age to 3 months postpartum. |
| 13 | Y.Sun et al. (2021) (42) | Mindfulness | The mindfulness training program contained eight sessions, each composed of a thematic curriculum and formal and informal training lasting for 1 week. Formal mindfulness training included body scan, mindful breathing, mindful stretching, and mindful meditation lasting 15-25 minutes per day. Informal training was also recommended to be practiced every day, including pausing during daily life, mindful eating, mindful walking, and 3-minute breathing practices | Pregnant women received 8-week regular WeChat health consultations. | Formal and informal mindfulness training via a custom-built mobile app | Nursing assistant with experience in prenatal care | **8-week** mindfulness training |
| 14 | Wang et al. (2023) (43) | Mindfulness | The course mainly comprised raisin meditation, breathing awareness, body scan, mindful yoga and meditation, labor pain cognitive education, and pain management withholding ice exercises | Received an online childbirth education course with the recorded video by the WeChat applet for 21 days. | Direct instruction in the hospital and practice at home | Two Mindfulness-Based Childbirth and Parenting teachers | A 4-day weekend for four consecutive weeks, (Total 14 hours) |
| 15 | Leng et al. (2023) (44) | Mindfulness | The program focused on loving-kindness, compassion, appreciative joy, and equanimity meditations. The application was designed with two components: An app module self-learning for meditation practice and a Group chat between therapists who answered questions and concerns from participants. | The web-based perinatal education program contained maternal and child health care and common postpartum discomfort and care. | Online invention using applications to deliver prevention program | Application and psychotherapist | Online self-learning with eight weekly sessions, and the therapist responded to the questions weekly. |
| 16 | Hassdenteufel et al. (2023) (45) | Mindfulness | The intervention consisted of eight weekly sessions involving psychoeducational and obstetrical content, mindfulness exercises, and cognitive behavioral approaches.  The psychoeducational content encompassed the occurrence of pregnancy-related stress, the emergence of vicious mental circles, and individual sources of strength. Meditated skills comprised how to exit from the vicious circle of fear and the use of mindful breathing and mindful body scans. | Care as usual | The application delivered the online intervention. | The application was designed in several forms, such as audio files, videos, written content, a personal skills box, and interactive worksheets | 45 min/session x 8 session. |
| 17 | Pan et al. (2019) (46) | Mindfulness | The intervention taught participants how to monitor their sensory and emotional states and cognitive processes, deepen their sensory self-awareness, and become more mindful of the process of labor and parenting. The program used formal and informal mindfulness to cope with the stressful events of pregnancy, childbirth, and the postpartum period. | The standard presentation on physiological and psychological information and practice skills during pregnancy and after childbirth | Direct intervention for group pregnant women. | The first author of the article | 3 hours/week x 8 weeks. |
| 18 | Guo et al. (2020) (47) | Mindful Self-Compassion | The intervention focused more on self-compassion, the pressure of being a new mother, and the incapability of self-regulation.  The new episode was opened the next day if pregnant women completed the prior one. | Care as usual | Online intervention | Website | Total 10 hours of training for 36 episodes within 6 weeks |
| 19 | Arakawa et al. (2023) (48) | mHealth consultation | The consultation service provided general advice by medical specializations to address health-related concerns and shared preventive care knowledge for mental health problems.  Online services were available in many methods, such as voice calling, text messaging/chat, and video calling.  The service provided consultation during pregnancy and childbirth. | Information on pregnancy and childcare is available on a website. | Online platform  - Line platform for the intervention group.  - Website for control group | Obstetrician-gynecologists, Pediatricians Midwives | 10-minute consultation/ time.  - women can book as many times as possible.  From prenatal until postnatal time. |
| 20 | Cordero et al. (2018) (49) | Exercise | Moderate physical exercise in an aquatic environment from weeks 20 to 37 of gestation.  Aerobic session followed by strength and endurance exercises and final stretching and relaxation | Activities as usual | Direct | No information | One hour per time. Three times per week.  Duration: 17 weeks. |
| 21 | Navas el al. (2021) (50) | Exercise | The intervention included standard antenatal care and aquatic aerobic exercise.  The aquatic aerobic exercise program was designed so that each woman maintained an estimated heart rate of 55–65% of the maximum (140 bpm). | Standard antenatal care | Direct instruction | Midwives | 45 minutes exercise x  3 times/week in an indoor pool for 5 months during pregnancy. |
| 22 | Coll et al. (2019) (51) | Exercise | The moderate exercise consisted of aerobic activities, strength training, and pregnancy-specific floor exercises.  Three pregnant women were trained by one instructor. | Maintaining their usual daily activities | Direct instruction | Exercise instructors with at least one year of postgraduate experience. | 60 minutes 3 times per week, lasting 16 weeks during pregnancy. |
| 23 | Mohammadi et al. (2015) (52) | Exercise | The theoretical and practical education about the importance of exercise  during pregnancy and instruction of exercise at home. The exercises were low-intensity stretching and breathing  practices.  Intervention group 1: recommended to home-exercise until delivery  Intervention group 2: recommended to to home-exercise 2 month postnatal exercises. | Antenatal and postnatal ordinary education in a 40 min session | Direct | Researchers work at the Midwifery Department. | Three times per week, 20- 30 minutes each. |
| 24 | Shimpuku et al. (2022) (53) | Education program | The intervention taught pregnant women to understand newborn sleep-wake cycles and a baby’s states (Resting/Ready/ Rebooting “Zones”) and to respond to a baby’s stress response (changes in skin color, movement of breathing, gaze aversion)  Participants learned and practiced safe swaddling. | Care as usual | Direct class with groups of pregnant women.  Video, the *roadmap* handout, and an appropriate cloth for swaddling were provided. | Researcher team | Two hours before delivery. |
| 25 | Zhao et al. (2021) (54) | Education program | Four sessions of the intervention focused on teaching pregnant women about maternal mood, anxiety, and depression and their management skills; explaining the preparation of giving birth: onset, process, and pain management; providing PPD information and the solution and coping skills for managing PPD; breastfeeding. | Usual perinatal care and routine group education courses in the hospital about maternal health and childcare. | Direct class with groups of pregnant women | Midwives with the training of psychology by psychiatrist and lactation consultant. | 4 sessions x 60 min/session.  4 sessions on 4 antenatal examination appointments. |
| 26 | Mohammadi et al. (2021) (55) | Education program | Based on Bandura's self-efficacy theory.  The training program encompassed the following components of self-efficacy:  1. Attention to physiological states.  2. Performance attainment: Training included personal hygiene, awareness of pelvic anatomy, practical demonstration of pregnancy and delivery exercises, relaxation techniques, adaptation to physiological changes, dealing with pregnancy complications, nutrition, mental health, coping with labor pain, and stages of delivery.  3. Vicarious experiences: Mothers received shared experiences from those who have had vaginal deliveries and expertise in parenting.  4. Verbal persuasion: Mothers were encouraged to exercise and relax at home.  5. Attention to emotional states. | Routine prenatal cares | Direct class with groups of pregnant women. | Trained midwife with a bachelor’s degree in midwifery.  CD training was given to mothers | 8 sessions x 2 hours/session from 20^th^ week of pregnancy. |
| 27 | Beydokhti et al. (2021) (56) | Education program | Following PRECEDE–PROCEED model.  Content of the the training: anatomical and physiologic changes, prenatal and postnatal care, mental health during and after pregnancy, events during pregnancy and after childbirth, maternal feelings and thoughts, and postpartum issues, and their solutions. | Routine prenatal care | Direct class with a group of pregnant women | the research team | 4 sessions x 60-90 minutes/session |
| 28 | Scorza et al. (2020) (57) | Education and mindfulness program | The intervention program consisted of three sessions: 1) Mindfulness and self-reflection skills and 2) Parenting skills. 3)Psychoeducation | Care as usual with enhanced support for finding treatment | Direct class individually | Psychologist at PhD level | 3 sessions with 150 minutes in total and one motivational interviewing |
| 29 | Moshki et al. (2014) (58) | Education program | Following the health locus of control theory (HLC).  The program's content covered anatomic and physiological changes, nutrition, common complications during pregnancy, mental health and communication skills, pregnancy stages, delivery and pain reduction methods, postpartum health, emotions and attitudes of women with a focus on components of HLC (internal HLC, powerful others HLC, chance HLC) | Routine care available at health centres | Workshop | No information | 3 workshops with 4 hours/ workshop |
| 30 | Zhao et al. (2019) (59) | Education program | The intervention program consisted of 5 sessions focusing on maternal mental health for pregnant women with complications of pregnancy and one session for their husband. The sessions covered the identification of anxiety and depression during pregnancy, knowledge enhancement of high-risk pregnancy, understanding postpartum depression, coping with family relationship issues and stressful events, discussing delivery experiences, and instructing husbands to identify and respond to postpartum depression symptoms. | Care as usual | Direct class with a group of pregnant women | Clinical psychologists and senior obstetric head nurses. | 6 sessions with 2 hours per session. |
| 31 | Haga et al. (2019) (60) | Education program | Usual care and the web-based program called Mamma Mia.  The "Mamma Mia" program includes materials and tools such as mindfulness and relaxation activities, guidance on communicating with your partner or healthcare provider, planning for breastfeeding, and bonding with your baby.  Each session is designed to take about 10 min and must be completed before users can move on to the next session. | The usual perinatal care in Norway includes eight consultations during pregnancy and six consultations during the six months following birth. | Automated web-based intervention by email and interactive websites, combining text, pictures, prerecorded audio files, and user input. | Web-based automatically | Total 44 sessions over 11.5 months divided into 3 phases, starting from the second trimester until 6 months postpartum. |
| 32 | Sanaati et al. (2018) (61) | Education program | Two groups: group 1 was pregnant women and their husbands; group 2 was pregnant women only and group 2.  The lifestyle-based training during pregnancy included educational content on sleep hygiene, nutrition, physical activity and exercise, self-image, and sexual matters. | The usual care | Direct class with groups of pregnant women and their husbands. | PhD student in reproductive health and psychiatric nursing. | 4 times x 60-90 minutes/time |
| 33 | Zhao et al. (2018) (62) | Education program | The intervention was implemented for first-time parents, including 5 sessions for pregnant women and one session for their husbands.  Topics of the sessions were increasing awareness of antenatal anxiety and depression, improving knowledge about high-risk pregnancy, understanding postpartum depression and its identification, coping skills for family conflicts, supporting psychological adjustment during delivery, and helping husbands identify their wives’ postpartum depressive symptoms and provide their support. | Routine prenatal care in China. | Direct class with a group of pregnant women and their husbands | The research team. | 6 sessions x 90 minutes per session |
| 34 | Collado et al. (2014) (63) | Education program | Received a novel psychosomatic antenatal program using the Tourné psychosomatic approach focusing on body awareness sensations, construction of an individualized childbirth model, preparation for parenting and attachment | The standard model of antenatal education | Direct class by a small group | Nurse facilitator | Two hours/session, weekly during the second trimester |
| 35 | Cauli et al. (2019) (64) | Education program | The multidisciplinary psychosocial interventions (MPI) included screening, diagnostic assessment, and interventions**.** The intensity varied according to the level of risk of postpartum depression (PPD) and had specific aims. High-risk women were offered 12–24 weekly interpersonal psychotherapy sessions and psychiatric monitoring. Low-risk women were offered psychosocial counseling. No-risk women were offered an educational intervention of primary prevention. | No control goup | Direct individual intervention. | a psychiatrist and two clinical psychologists | High risk group: 12-24 weekly  Low-risk group: 3/4 sessions – 50 min  No risk: 1 hour |
| 36 | Shorey et al. (2019) (65) | Education program | Received the Supportive Education for Parents Program (SEPP) in addition to the standard routine perinatal hospital care. The SEPP adopted a 3-step approach: a 30-min telephone-based antenatal educational session, a 60-min telephone-based immediate postnatal educational session, and a mobile health (mHealth) app follow-up educational session made available for 4 weeks postpartum | Received routine perinatal care provided by the hospital | Telephone-based educational sessions, mobile health app | Research Assistant, trained midwife | A 90-min telephone-based perinatal educational session, 4 weeks postpartum follow-up by mHealth app |
| 37 | Çankaya et al. (2020) (66) | Educational program | Received antenatal education based on Content was based on Dick-Read’s “natural birth”, Lamaze’s “psychoprophylaxis”, Balaskas’s “active birth”, and Mongan’s “hypnobirthing” philosophy.  Topics included childbirth philosophies, birth fear, and trust/control exercises | Received prenatal care service routinely provided at the polyclinics of the same hospital | Direct class by a small group (8-10 people) | Not specified. | Twice a week for 2 weeks (total 16 hours) |
| 38 | Ahmadpour et al. (2022) (67) | Educational program | Received interventions based on the mother’s requested birth plan, which included the mother’s preferences in labor, mobility, eating and drinking, monitoring, pain relief, drug options, labor augmentation, pushing, amniotomy, episiotomy, infant care, and caesarean section. | Received routine hospital care | Direct class individually | Principal investigator (PhD student of Midwifery) under the supervision of an obstetrician | Two face-to-face sessions |
| 39 | Shorey et al. (2023) (68) | Psychoeducational program | Received the Supportive Parenting App (SPA) intervention, a mobile app–based psychoeducation and peer support program to support parents from pregnancy to 6 months postpartum. Participants were given access to the SPA and contacted by their assigned peer volunteer shortly after recruitment. The SPA included knowledge-based content curated by a multidisciplinary team on various topics and was presented in written articles, audio clips, and videos. Discussion forums, FAQs, and expert advice were also available. | Received only the standard care provided by the hospital | Mobile app with written articles, audio clips, videos, discussion forums, expert advice, peer support | A multidisciplinary team of health care professionals, trained peer volunteers | From 24 weeks of gestation to 6 months postpartum |
| 40 | Nakajima et al. (2023) (69) | Education program | Participated in a pregnancy program to strengthen the relationship between husband and wife in addition to the regular maternity class. Contents included information on the physical and mental  risks of older primiparas and husbands' support, couple discussion on postpartum couple relationships, interaction among participating couples and couples' pair stretches | Participated only in the normal maternity class. | Direct class with groups of pregnant women and their husbands. | Principal investigator and co-principal investigator (licensed midwife) | Once during pregnancy for 2 hours on weekend |
| 41 | Ahrne et al. (2023) (70) | Education program | Received language-supported group antenatal care (gANC), a combination of gANC and individual check-ups, with language support and integrated childbirth and parenting education.  Group sessions cover lifestyle, pregnancy, birth, practical birth preparations, the newborn baby, breastfeeding (and alternatives), parenthood and relationships. Language support was provided by a female interpreter in every group session | Received standard, midwifery-led individual care in accordance with Swedish national guidelines | Direct group sessions, individual appointments by language interpretation | Midwives and a female interpreter (trained nurse assistant) | Seven 60-minute group sessions, plus 15-minute individual appointments |
| 42 | Khodadad et al. (2021) (71) | Supplement | The intervention group received two 40 mg pills of vitamin B6 daily from the 28th week before delivery and one 40 mg pill of vitamin B6 for one month postpartum.  Researchers reminded participants to take Vitamin B6 every two weeks. | The control group received a placebo. | Directly provided at the health center. | Researchers | From the 28^th^ week of pregnancy to one month postpartum. |
| 43 | Vaz et al. (2017) (72) | Supplement | The intervention group received fish oil capsules containing a total daily dose of 1.8 g of n-3 (1.08 g of EPA and 0.72 g of DHA). Participants took 6 capsules daily for 16 consecutive weeks.  Women were advised to return all empty supplement packages at every visit, and a research assistant estimated the number of capsules they had taken during the interval. | The control group received a placebo, which is a similar package to fish oil capsules. | Directly provided at the health center. | Researchers. | 16 consecutive weeks started from the 3rd trimester. |
| 44 | Sousa et al. (2023) (73) | Supplement | Received two Fish oil capsules a day offered 260 mg of EPA (eicosapentaenoic acid) and 1440 mg of DHA. | Received olive oil capsules | Received detailed guidance on how to use and store the supplement | Not specified. | Supplementation lasted until childbirth (approximately 16 weeks) |
| 45 | Kianpour et al. (2018) (74) | Inhalation Aromatherapy | 7 drops of lavender essential oil and 1 cc of rose water at a concentration of 100% were put on the cloth. They put the cloth on their mouths And inhaled 10 deep breaths before sleeping and put them next to their pillows until morning. | 7 drops of odorless sesame seed oil and 1 cc of sweat musk at a concentration of 100% | Follow-up via phone call. | Researchers | from the 38th week of pregnancy until 6 weeks after delivery |
| 46 | Zlotnick et al. (2016) (75) | Interpersonal therapy | Small groups of pregnant women were trained in psychoeducational components and interpersonal therapy for improving relationships and building social support.  It focused on managing role transitions, developing a support system, improving communication skills, setting goals, and providing psychosocial resources for new mothers. | Antenatal care routine in the US. | Small pregnant women groups (2-5 women) | A registered nurse and two trainers with bachelor's degrees. | 4 times x 90min/time and 50 min individually booster after delivery 2 weeks. |
| 47 | Phipps et al. (2013) (76) | Interpersonal therapy | Participants received the Project REACH intervention. Central features of the intervention were psychoeducation, skills training (e.g., problem-solving, behavioral activation, communication analysis), and interpersonal process (e.g., role-playing, communication analysis). Participants received the book *Baby Basics: Your Month-by-Month Guide to a Healthy Pregnancy* | Participants in the control condition used the *Baby Basics* book.. | Direct class with groups of primiparous pregnant adolescents. | Not specified. | Five one-hour prenatal sessions with a postpartum booster session |
| 48 | Phipps et al. (2020) (77) | Interpersonal therapy | Participants received the REACH intervention program [Phipps 2013] for primiparous and multiparous adolescents | Participants in the control condition used the *Baby Basics* book | Direct class with groups of pregnant women | Paraprofessionals | Five individual 1-hour prenatal sessions with an in-hospital postpartum booster session |
| 49 | Ngai et al. (2022) (78) | Interpersonal therapy | Received couple-based interpersonal psychotherapy**.** Common IPT techniques, namely communication analysis and role-playing, were used | Received usual care | Direct couples attended the antenatal sessions together in groups of 8–10 | Experienced midwives | Three weekly 2-hour antenatal sessions and two 30-minute telephone follow-up sessions. |
